# Albumin administration and 28-day mortality in sepsis-induced myocardial injury: a propensity score-matched analysis

**DOI:** 10.3389/fphar.2026.1844892

**Published:** 2026-06-30

**Authors:** Qi-Fang He, Ge Zhang

**Affiliations:** Department of Critical Care Medicine, Sir Run Run Shaw Hospital, Zhejiang University School of Medicine, Hangzhou, Zhejiang, China

**Keywords:** albumin, intensive care unit, MIMIC-IV database, mortality, septic-induced myocardial injury

## Abstract

**Background:**

Current evidence on the therapeutic effects of albumin administration in critically ill patients with sepsis-induced myocardial injury (SIMI) remains insufficient. To address this knowledge gap, we performed a retrospective cohort study to examine the association between albumin supplementation and key clinical outcomes in this patient population.

**Methods:**

This study included adult patients with SIMI who were treated in the intensive care unit (ICU). Data were extracted from the Medical Information Mart for Intensive Care IV (MIMIC-IV) database (version 2.2). Patients were categorized into two groups based on whether they received albumin infusion during their ICU stay: the albumin group and the non-albumin group. The primary endpoint was all-cause 28-day mortality. Kaplan-Meier (KM) curves were constructed to estimate survival probabilities, and log-rank tests were used to compare survival distributions between the two groups. Subgroup analyses were performed to explore heterogeneity in treatment effects and to assess the association of albumin infusion with outcomes across different patient characteristics.

**Results:**

A total of 433 patients were included in the albumin infusion group and 1126 in the non-albumin infusion group. After 1:1 propensity score matching (PSM), 648 patients were included in the analysis (324 in each group). Following PSM, no statistically significant differences were observed in mortality outcomes between the albumin and non-albumin groups: 28-day mortality (HR = 0.97, 95% confidence interval[CI]: 0.57–1.64, p = 0.90), 60-day mortality (HR = 0.89, 95%CI 0.56–1.41, p = 0.63), 90-day mortality (HR = 0.89, 95%CI 0.57–1.39, p = 0.61), and in-hospital mortality (HR = 0.85, 95%CI 0.55–1.33, p = 0.49). Notably, the albumin group exhibited significantly prolonged ICU length of stay (9.06 [4.65, 16.72] vs. 5.65[2.76, 10.50] days, P<0.01) and hospital length of stay (22.56 [13.42, 34.05] vs. 14.50 [8.32, 22.36] days, P<0.01) compared to the non-albumin group.

**Conclusion:**

In this propensity score-matched cohort of critically ill patients with sepsis-induced myocardial injury, albumin-based resuscitation strategy demonstrated comparable 28-day survival outcomes to crystalloid-focused fluid management.

## 1 Introduction

Sepsis, a life-threatening organ dysfunction caused by a dysregulated host response to infection, remains one of the leading causes of mortality in ICUs (Singer et al., 2016). Among its complications, SIMI significantly exacerbates clinical outcomes by impairing cardiac function through multifactorial mechanisms (Bi et al., 2022), including apoptosis, mitochondrial dysfunction (Cimolai et al., 2015), dysregulated autophagy (Huang et al., 2024), excessive inflammation, oxidative stress (Chen et al., 2025), and pyroptosis (Jin et al., 2025). Concurrently, sepsis-induced degradation of the endothelial glycocalyx (eGC) contributes to vascular hyperpermeability, coagulopathy, and microcirculatory disturbances, further aggravating myocardial edema and heterogeneous tissue perfusion (L’Heureux et al., 2020).

Albumin has emerged as a potential therapeutic agent in this context, owing to its dual role in stabilizing the eGC and modulating systemic inflammation. By binding to glycocalyx polysaccharides, albumin enhances endothelial nitric oxide synthesis while exerting antioxidant, anti-inflammatory, and immunomodulatory effects (Zhan et al., 2025). Clinical evidence indicates that albumin supplementation decreases circulating levels of VE-cadherin, a biomarker whose reduced concentration has been correlated with improved clinical prognosis (Piotti et al., 2021). Notably, randomized trials demonstrate that 20% albumin administration rapidly improves microcirculatory parameters, as evidenced by superior capillary refill time normalization (63% vs. 29%, P = 0.02) and lactate clearance compared to saline (Gabarre et al., 2024). A retrospective analysis further revealed that early albumin infusion within 24 h was associated with significantly lower 28-day mortality in septic shock patients complicated by ARDS (34.8% vs. 48.1%, p = 0.031) (Wang et al., 2023).

Therapeutic efficacy appears concentration- and context-dependent. In critically ill patients with sepsis, comparative analysis demonstrated that administration of 5% human serum albumin (HSA) conferred superior survival benefits compared with 25% HSA (adjusted hazard ratio [HR]: 0.63; 95% CI: 0.54–0.73; p < 0.001) (Xu et al., 2022). In cirrhotic patients with septic shock, albumin demonstrates superior hemodynamic stabilization efficacy compared to normal saline, particularly in reversing refractory hypotension (Philips et al., 2021). A recent meta-analysis demonstrated that 20% albumin administration was associated with a significant 19% reduction in 90-day mortality risk among septic shock patients (pooled OR 0.81, 95% CI 0.67–0.98; p = 0.03), showing superior efficacy compared to both 4%–5% albumin solutions and crystalloid resuscitation fluids (Geng et al., 2023). A study has demonstrated that daily albumin infusion in cirrhotic patients with sepsis is associated with a reduced 28-day mortality risk (hazard ratio [HR] 0.76; 95% confidence interval 0.61–0.94). Within this population, the greatest clinical benefits were observed in patients meeting any of the following criteria: serum albumin concentrations between 2.5-3.0 g/dL, serum lactate levels ≥2 mmol/L, mean arterial pressure (MAP) < 60 mmHg, or vasopressor requirements equivalent to 0.2–0.3 μg/kg/min of norepinephrine (NEE) (Hu et al., 2023). Current international guidelines cautiously endorse albumin as an adjunct to crystalloids in sepsis resuscitation, albeit with weak recommendations due to heterogeneous evidence (Evans et al., 2021).

The impact of albumin administration on outcomes in patients with SIMI remains poorly characterized. To address this knowledge gap, we conducted a comprehensive retrospective cohort study evaluating the association between albumin administration and mortality risk in this patient population.

## 2 Methods

### 2.1 Database source

This retrospective cohort study utilized data from the MIMIC-IV database (version 2.2), a publicly available critical care repository containing de-identified clinical data of 73,181 ICU admissions at Beth Israel Deaconess Medical Center (2008–2019). The database encompasses comprehensive electronic health records including vital signs, laboratory measurements, medication administrations, and survival outcomes. The Institutional Review Board (IRB) at Beth Israel Deaconess Medical Center conducted ethical oversight of this study, granting an exemption for both informed consent requirements and authorization for subsequent data dissemination. The author successfully completed the mandatory CITI Program research ethics training (Certification ID: 48361544) and executed the PhysioNet data use agreement prior to obtaining database access.

### 2.2 Population criteria

The study population comprised adult patients (≥18 years) with ≥24-h ICU stays in the MIMIC-IV database. We applied the following sequential selection criteria:

Inclusion: 1. Fulfilled Sepsis-3 criteria with concurrent SIMI, defined as: 1) Sepsis is clinically defined as the presence of suspected infection accompanied by a Sequential Organ Failure Assessment (SOFA) score of two or higher (Singer et al., 2016). 2) Serum cardiac troponin T >0.01 ng/mL, the 99th percentile upper reference limit value for Troponin T, consistent with established diagnostic thresholds (Vallabhajosyula et al., 2017; Xu et al., 2023); 3) No alternative explanation for troponin elevation; 2. First ICU admission during hospitalization (excluding readmissions).

Exclusion: 1. Myocardial infarction; 2. Cardiomyopathy; 3. History of cardiac surgery (coronary artery bypass grafting, percutaneous coronary intervention, valve repair/replacement, atrial/ventricular septal repair); 4. Chronic heart failure; 5. Pulmonary embolism or cardiac arrest; 6. Missing baseline serum albumin levels.

### 2.3 Data processing

Data were extracted from the MIMIC-IV database using Structured Query Language via pgAdmin4. Baseline data for all variables were captured at the first recorded instance following admission to the Intensive Care Unit (ICU). The data encompass demographic characteristics including age, gender; vital signs and laboratory results including body temperature (T), respiratory rate (RR), systolic blood pressure (SBP), diastolic blood pressure (DBP), mean arterial pressure (MAP), heart rate (HR), oxygen saturation (SpO2), serum albumin, serum cardiac troponin T (cTnT), blood glucose, white blood count (WBC), platelet count (PLT), hemoglobin, lactate, blood urea nitrogen (BUN), creatinine, estimated glomerular filtration rate (e-GFR), potassium, prothrombin time (PT), suspected infection to antibiotic administration time; comorbidities such as chronic obstructive pulmonary disease (COPD), hypertension, chronic kidney disease (CKD), diabetes mellitus, liver cirrhosis, and malignant neoplasm; disease severity scores including Acute Physiology Score III (APS III) and SOFA score; interventions and treatments involving continuous renal replacement therapy (CRRT), vasopressors(including dopamine, epinephrine, norepinephrine, phenylephrine, vasopressin, dobutamine, milrinone), albumin infusion, and invasive mechanical ventilation (IMV). Key variables were defined as follows:early albumin administration as the first dose within 6 h of ICU admission (late: ≥6 h); albumin concentration as low (5%) or high (25%) based on the initial product; total cumulative dose, calculated as concentration × volume in grams, and dichotomized into low-dose (<75 g) or high-dose (≥75 g) groups based on the median; troponin T stratification with moderate/severe elevation defined as > 0.06 ng/mL and mild elevation as ≤ 0.06 ng/mL(based on the median); daily fluid balance for the first three ICU days as total intake minus total output; hospital-acquired infections as the identification of new pathogenic bacteria in cultures obtained >48 h after admission; and new-onset acute kidney injury (AKI) as AKI developing after the first 6 h of ICU stay in patients without AKI at baseline, according to KDIGO criteria.

### 2.4 Outcomes

The primary outcome was defined as 28-day mortality, with secondary endpoints encompassing 60- and 90-day mortality, in-hospital mortality, as well as ICU and hospital length of stay (LOS).

### 2.5 Statistical analysis

Data following normal distribution were analyzed with Student’s t-test and presented as mean and standard deviation, while non-normally distributed data were evaluated with the two-sample Wilcoxon rank-sum test (Mann–Whitney) and reported as median and interquartile range (IQR). Categorical variables were analyzed using Pearson’s chi-square test. The missing data rate was highest for lactic acid (13.3%), while other variables exhibited <10% missingness. Detailed documentation of missing values, abnormal measurements, and corresponding data handling procedures are provided in Additional file Supplementary Table A1.

### 2.6 Propensity score matching (PSM) analyses

We employed propensity score matching in two distinct phases. First, for the primary analysis comparing albumin versus no albumin administration, a 1:1 propensity score matching (PSM) with an absolute caliper of 0.05 (non-scaled) was performed using a comprehensive set of clinically relevant covariates (all listed in Table 1) to balance baseline characteristics between groups. Post-matching covariate balance was evaluated using standardized mean differences (SMD) and statistical hypothesis testing (P-values), with predefined criteria of SMD <0.1 and P > 0.05 indicating adequate balance. Subsequently, to address specific exploratory questions regarding albumin use, we conducted three additional PSM analyses comparing: (1) early vs. late timing, (2) 5% vs. 25% concentration, and (3) low vs. high total dose. For these exploratory analyses, we applied the same matching algorithm but used the refined set of covariates as detailed in the footnote of Table 1. The success of all matching procedures was evaluated using standardized mean differences (SMD <0.1 indicating adequate balance), with the balance for the primary and exploratory analyses visualized in Additional File 1: Supplementary Figures A1–A4.

**TABLE 1:** Basic characteristics.

Valuables	Original cohort			Propensity score matched cohort			​
	Non-albumin group (1,126)	Albumin group (433)	P value	Non-albumin group (324)	Albumin group (324)	P value	SMD
Gender (Male(%))	631 (56.0)	236 (54.5)	0.585	167 (51.5)	175 (54.0)	0.582	0.049
Age (y)	66.6 (16.5)	64.0 (15.2)	0.0041	65.1 (15.1)	65.0 (15.5)	0.920	0.008
Comorbidities, n (%)							
Hypertension, n (%)	585 (52.0)	239 (55.2)	0.251	182 (56.2)	174 (53.7)	0.580	0.050
Diabetes, n (%)	392 (34.8)	165 (38.1)	0.224	121 (37.3)	114 (35.2)	0.624	0.045
COPD, n (%)	81 (7.2)	40 (9.2)	0.177	22 (6.8)	28 (8.6)	0.462	0.069
CKD, n (%)	320 (28.4)	119 (27.5)	0.713	80 (24.7)	90 (27.8)	0.422	0.070
Cirrhosis, n (%)	115 (10.2)	163 (37.6)	0.000	78 (24.1)	90 (27.8)	0.324	0.085
Malignant neoplasm, n (%)	316 (28.1)	133 (30.7)	0.300	104 (32.1)	102 (31.5)	0.933	0.013
Vital signs							
T (°C)	36.8 (0.9)	36.5 (1.1)	0.0000	36.6 (1.0)	36.6 (1.0)	0.857	0.014
RR (insp/min)	20.7 (6.0)	20.2 (6.1)	0.1272	20.1 (5.9)	20.3 (5.8)	0.575	0.044
SBP (mmHg)	124.7 (26.5)	118.3 (25.2)	0.0000	118.9 (25.3)	120.5 (25.7)	0.428	0.062
DBP (mmHg)	68.3 (19.7)	64.0 (18.1)	0.0001	64.7 (18.7)	65.3 (18.2)	0.685	0.032
MAP (mmHg)	92.5 (21.0)	93.2 (21.7)	0.5319	94.2 (21.9)	93.0 (21.5)	0.478	0.056
SpO2 (%)	98 [95,100]	98 [95,100]	0.4016	98 [95.8, 100]	98 [95, 100]	0.484	0.060
Critical score							
SOFA score	3 [2,5]	4 [3,6]	0.0000	4 [2, 6]	4 [2, 6]	0.841	0.005
APS III	52 [41,66]	61 [49,76]	0.0000	57 [46, 73]	58.5 [46, 75]	0.997	0.031
Baseline laboratory measurements							
Albumin (g/dL)	3.0 (0.6)	2.8 (0.7)	0.0000	2.8 (0.6)	2.8 (0.7)	0.142	0.115
cTnT (ng/mL)	0.06 [0.03,0.16]	0.06 [0.03,0.14]	0.2402	0.05 [0.03, 0.16]	0.06 [0.03, 0.15]	0.819	0.031
Glucose (mg/dL)	133.5 [107,178]	133 [106,177]	0.5207	131.5 [106.8, 170]	135.5 [106, 178.3]	0.529	0.020
K (mEq/L)	4.3 (0.9)	4.3 (0.8)	0.8884	4.2 (0.9)	4.3 (0.8)	0.743	0.026
BUN (mg/dL)	28 [18,47]	26 [16,43]	0.1114	25 [17.8, 39]	26 [16, 43]	0.843	0.057
Lactate (mmol/L)	1.7 [1.3,2.5]	2.3 [1.5,3.8]	0.0000	1.9 [1.4, 3.1]	2.1 [1.4, 3.5]	0.312	0.022
Creatinine (mg/dL)	1.3 [0.9,2.3]	1.2 [0.9,2.1]	0.1516	1.2 [0.8, 1.8]	1.2 [0.9, 2.1]	0.499	0.046
e-GFR	50.5 [26.8,81.6]	54.4 [28.6,83.4]	0.1495	56.3 [32.4, 88.1]	54.7 [28.4, 84.9]	0.563	0.023
PT(s)	14.5 [12.9,17.2]	16.5 [14,20.4]	0.0000	15.7 [13.3, 19.4]	15.9 [13.7, 19.4]	0.299	0.002
WBC (K/uL)	12.3 [7.9,17.2]	11.8 [7.6,16.9]	0.2674	11.6 [7, 17]	12 [8.1, 17.2]	0.478	0.018
Hemoglobin (g/dL)	10.8 (2.3)	10.2 (2.2)	0.0000	10.4 (2.3)	10.4 (2.2)	0.976	0.002
PLT (K/uL)	194 [132,269]	154 [102,243]	0.0000	172 [118, 250]	174 [115, 259]	0.944	0.001
Time to antibiotics (hr)	8 [4,17]	9 [3,24]	0.2754	8 [4, 22]	8 [3, 22.3]	0.397	0.081
Treatment							
Vasopressors	520 (46.2)	329 (76.0)	0.000	227 (70.1)	231 (71.3)	0.796	0.027
IMV	603 (53.6)	334 (77.1)	0.000	241 (74.4)	240 (74.1)	1.000	0.007
CRRT	88 (7.8)	88 (20.3)	0.000	41 (12.7)	45 (13.9)	0.728	0.036

Data are presented according to variable type and distribution. Continuous variables are expressed as mean (SD) for normally distributed data (compared using the independent samples t-test) or as median [IQR] for non-normally distributed data (compared using the Mann-Whitney U test). Categorical variables are expressed as number (percentage) and were compared using the Chi-square test. Abbreviations: SD, standard deviation; IQR, interquartile range.

### 2.7 Outcome analyses

The primary outcome, 28-day mortality, was analyzed using Kaplan-Meier survival curves with the log-rank test. Univariable Cox proportional hazards regression was employed to estimate hazard ratios (HRs) with 95% confidence intervals (Additional file 1: Supplementary Table A2). Furthermore, exploratory analyses utilizing the same Cox model were conducted to assess the association between 28-day mortality and albumin administration characteristics—specifically timing (early vs. late), concentration (5% vs. 25%; low vs. high concentration), and total cumulative dose (low vs. high, dichotomized at the median)—within the cohort of patients who received albumin. Finally, stratified subgroup analyses were further conducted to assess the heterogeneity of the albumin infusion effect across clinically relevant patient categories.

### 2.8 Sensitivity analyses

To evaluate the robustness of our primary findings, we performed two sensitivity analyses: propensity score matching on datasets that sequentially excluded patients with missing lactate values only and subsequently those missing both lactate and prothrombin time, followed by comparative mortality assessments (Additional file 1: Supplementary Table A3); and logistic regression analysis incorporating multiple imputation for missing data to evaluate the mortality difference between groups (Additional file 1: Supplementary Table A4).

A two-tailed P-value threshold of 0.05 was established as the criterion for statistical significance in all analyses. All statistical analyses were conducted using Stata 17.0 and R 4.4.2.

## 3 Results

### 3.1 Baseline characteristics

**FIGURE 1:**
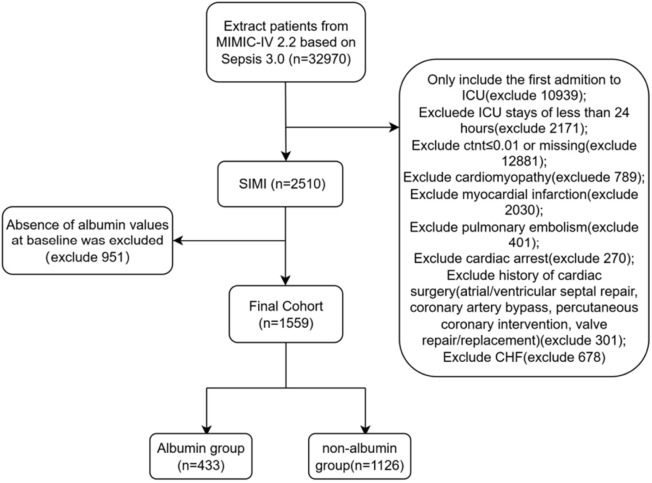
Flowchart.

The study cohort selection process is detailed in Figure 1, with 1,559 patients ultimately included from an initial pool of 32,970 after applying exclusion criteria. Baseline characteristics before and after propensity score matching are summarized in Table 1. We compared the absolute standard deviation between the original cohort and the matched cohort, and all baseline features were well balanced after PSM (Table 1; Additional file Supplementary Figure A1).

### 3.2 Primary outcome

**FIGURE 2:**
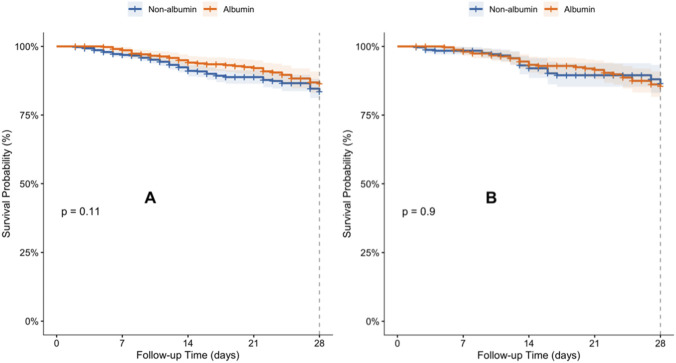
Kaplan-Meier curve for 28-day mortality **(A)** Original Cohort; **(B)** Propensity Score Matched Cohort).

No significant difference in 28-day mortality was observed between the albumin infusion group and the control group (P = 0.11), and this non-significant association remained after PSM (HR = 0.97, 95%CI: 0.57–1.64, p = 0.90) (Figure 2, Table 2). The robustness of this finding was further validated through sensitivity analyses: after sequential exclusion of cases with missing lactate or prothrombin time values and subsequent iterative propensity score matching, no significant mortality differences were observed between groups (Additional file Supplementary Table A3); similarly, logistic regression using multiply imputed lactate and PT data, adjusted for the same covariates, showed a comparable mortality risk (adjusted OR = 1.07, 95% CI: 0.66–1.72; p = 0.791) (Additional file Supplementary Table A4). Collectively, these results reinforce the consistency of findings across methodological variations, confirming that albumin administration was not associated with reduced 28-day mortality in this study population.

**TABLE 2:** Association between albumin infusion and 28-day, 60-day, 90-day, in-hospital mortality in SIMI patients.

Outcomes	HR	CI
Primary outcome	​	​
28-day mortality	0.97	0.57–1.64
Secondary outcomes		
60-day mortality	0.89	0.56–1.41
90-day mortality	0.89	0.57–1.39
In-hospital mortality	0.85	0.55–1.33

### 3.3 Secondary outcomes and hospitalization course

Following PSM, although hazard ratios (HRs) consistently favored the albumin group, no statistically significant differences were detected in 60-day mortality (HR = 0.89, 95% CI 0.56–1.41), 90-day mortality (HR = 0.89,95% CI 0.57–1.39), or in-hospital mortality (HR = 0.85, 95% CI 0.55–1.33) between the albumin and control groups (Table 2).

Notably, both the original and PSM-adjusted analyses revealed significantly prolonged ICU stays (P<0.01) and hospital stays (P<0.01) in the albumin group compared to the non-albumin group (Table 3).

**TABLE 3:** Outcomes of original cohort and matched cohort.

Outcomes	Original cohort		P value	Matched cohort		P value
	Non-albumin (n = 1126)	Albumin (n = 433)		Non-albumin (n = 324)	Albumin (n = 324)	
28-day mortality (n,%)	91 (8.1)	42 (9.7)	0.11	24 (7.4)	33 (10.2)	0.90
60-day mortality (n,%)	110 (9.8)	55 (12.7)	0.03	32 (9.9)	45 (13.9)	0.63
90-day mortality (n,%)	113 (10.0)	58 (13.4)	0.03	33 (10.2)	47 (14.5)	0.61
In-hospital mortality (n,%)	117 (10.4)	60 (13.9)	0.01	34 (10.5)	49 (15.1)	0.49
ICU stay (days)	4.20 [2.29, 8.35]	9.66 [4.89, 16.78]	<0.01	5.65 [2.76, 10.50]	9.06 [4.65, 16.72]	<0.01
Hospital stay (days)	11.94 [7.04, 20.61]	22.79 [14.12, 35.04]	<0.01	14.50 [8.32, 22.36]	22.56 [13.42, 34.05]	<0.01

Association of Albumin Dosing Patterns with Clinical Outcomes.

As shown in Table 4, neither albumin timing (early vs. late), concentration (low vs. high), nor total dose (low vs. high) was significantly associated with 28-day mortality.

**TABLE 4:** Association between albumin administration characteristics and 28-day mortality in propensity score-matched Cohorts (Cox proportional hazards models).

Variable	Comparison groups	Hazard ratio	CI
Albumin administration timing	Early vs Late	2.21	0.95–5.12
Albumin concentration	Low vs High	0.70	0.30–1.62
Albumin total Dose	Low vs High	1.14	0.49–2.64

The propensity score-matched analyses (timing, concentration, dose) utilized the following covariates: age, gender, T, MAP, SBP, DBP, RR, SpO2, cirrhosis, diabetes, CKD, COPD, hypertension, and malignancy neoplasm, troponin T, albumin, e-GFR, lactate, SOFA, score, APSIII.

### 3.4 Fluid balance and safety outcomes

As shown in Table 5, the albumin group had a higher but non-significant positive fluid balance on day 1(3444 [1300, 7501] vs. 2995[1300, 5881], p = 0.096), followed by significantly higher balances on days 2(1808[437, 3297] vs. 1099 [-16, 2591], p<0.01) and 3(844 [-432, 2227] vs. 213 [-385, 1550], p = 0.005) compared to the non-albumin group. Both hospital-acquired infections(32.4% vs. 23.8%, p = 0.018) and new-onset acute kidney injury(21.9% vs. 13.3%, p = 0.005) occurred at significantly higher rates in the albumin group. Overall, albumin administration was associated with increased risks of infectious and renal complications in this cohort.

**TABLE 5:** Comparison of fluid balance and safety outcomes between albumin and non-albumin groups.

Variable	Albumin group (n = 324)	Non-albumin group (n = 324)	P-value
Fluid balance, median [iqr] (ml)			
Day 1	3444 [1300, 7501]	2995 [1308, 5881]	0.096
Day 2	1808[437, 3927]	1099 [-16, 2591]	<0.01
Day 3	844 [-432, 2227]	213 [-385, 1550]	0.005
Complications, n (%)			
Hospital-acquired infections	105 (32.4)	77 (23.8)	0.018
New-onset AKI after ICU admission	71 (21.9)	43(13.3)	0.005

## 4 Subgroup analysis of the primary outcome

**FIGURE 3:**
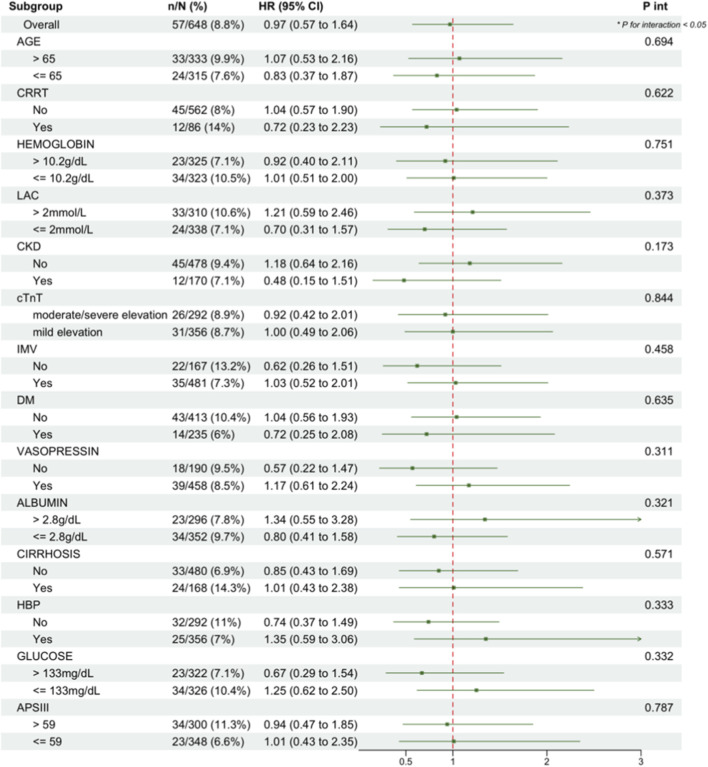
Forest plot of subgroup analysis of relationship between groups and 28-day mortality. Note: Hazard ratios (HRs) and 95% confidence intervals (CIs) were derived from Cox proportional hazards models. The number of events (n) and total participants (N) in each subgroup are shown as n/N (%). The vertical dashed line indicates HR = 1 (no effect). Squares represent point estimates, and lines indicate 95% CIs. P values for interaction test whether the effect varies across subgroups; asterisks denote P for interaction <0.05.

Subgroup analyses demonstrated no statistically significant mortality reduction at 28 days with albumin administration in any subgroups. These included subgroups of patients with cirrhosis (P for interaction = 0.571) and chronic kidney disease (CKD, P for interaction = 0.173), and those stratified by cardiac injury severity based on troponin T levels (P for interaction = 0.844). Conditions such as cirrhosis and CKD were of particular interest due to their impact on albumin metabolism, while troponin stratification addressed potential differential effects based on the degree of myocardial injury. All other interaction p-values were >0.1. In the severe hypoalbuminemia cohort (baseline serum albumin ≤2.8 g/dL), a numerical trend toward improved survival was observed with albumin supplementation; however, this effect did not attain statistical significance(HR 0.80, 95%CI 0.41–1.58) (Figure 3).

## 5 Discussion

In the propensity score-matched cohort of SIMI patients with comparable baseline albumin levels (mean 2.8 g/dL in both groups; p = 0.142), albumin administration demonstrated no mortality benefit across all evaluated endpoints: 28-day, 60-day, and 90-day mortality, as well as in-hospital mortality. While landmark trials such as the SAFE study in general ICU patients (Finfer et al., 2004) and investigations in septic cancer patients (Park et al., 2019) have demonstrated equivalent mortality between albumin and crystalloid resuscitation, the specificity of these findings to the SIMI population remained unclear. Our subgroup analyses, which stratified by disease severity, comorbidities, and treatment protocols, echo this pattern of neutral treatment effects (all interaction p values >0.1). These preliminary findings suggest that albumin were not associated with a mortality benefit in the SIMI population, though this requires confirmation in future prospective studies.

Our analysis revealed that albumin administration was associated with substantially prolonged ICU stays (median 9.06 vs 5.65 days, p < 0.01) and extended total hospitalization durations (median 22.56 vs 14.50 days, p < 0.01). Patients receiving albumin demonstrated significantly greater positive fluid balance, higher rates of hospital-acquired infections, and a higher incidence of new-onset acute kidney injury. Given the observational design, we cannot determine whether the prolonged ICU stay is directly attributable to these complications, reflects residual patient selection bias, or represents a direct harmful effect of albumin infusion. Of these, residual selection bias is a less likely primary explanation given the propensity score matching, while a direct harmful effect of albumin constitutes a particularly important hypothesis that warrants further investigation focused on non-mortality outcomes and safety. The significantly higher complication rates in the albumin group suggest that these complications likely contribute to the prolonged stay, although the observational design precludes definitive determination of causality or exclusion of residual patient selection bias. These findings differ from a previous retrospective study by Liu C et al. (2021), which may be explained by differences in patient populations, albumin administration timing, or the higher rates of fluid retention and complications observed in our cohort.

Clinical evidence has revealed significant heterogeneity in albumin’s therapeutic efficacy during sepsis management across diverse patient populations. Some studies have reported potential survival benefits in specific subgroups, such as patients with septic shock complicated by AKI (Ge et al., 2021), sepsis-induced ARDS (Wang et al., 2023), or cirrhotic patients with septic hypotension (Philips et al., 2021). Conversely, a randomized trial comparing 5% albumin to balanced crystalloids observed numerically higher mortality in the albumin group (Gray et al., 2024). However, a recent meta-analysis (Bai et al., 2024) demonstrated no significant mortality benefit for albumin across multiple endpoints: overall (RR = 1.02, P = 0.56), ICU (RR = 1.06, P = 0.65), in-hospital/28-day (RR = 1.01, P = 0.68), and 90-day mortality (RR = 1.01, P = 0.65). Intriguingly, a retrospective study (Zhou et al., 2021) evaluating early resuscitation strategies in sepsis revealed that while albumin-crystalloid combination therapy (initiated within 24 h of crystalloid administration) was independently associated with extended ICU stays (10.72 days vs. 8.24 days; P < 0.001) - consistent with our result - while concurrently demonstrated an unexpected survival advantage at 28 days (increased survival: 3.39 days, 95% CI 2.53–4.25; P < 0.001). This mortality benefit discordance with our findings underscores the critical need for risk-stratified analyses in heterogeneous sepsis populations.

This investigation has several limitations requiring careful interpretation. First, the retrospective observational design inherently carries risks of selection bias and residual confounding, despite our application of propensity score matching to address measurable confounders. Second, the absence of randomized intervention allocation prevents definitive conclusions from our findings regarding the association between albumin administration and extended ICU hospitalization. Third, all data were derived from a single institution (the MIMIC-IV database), which restricts external validity and generalizability. Fourth, important unmeasured confounders related to the clinical decision to administer albumin may remain. The database lacks granular data on specific clinical triggers for albumin use (e.g., response to initial fluid resuscitation, dynamic hemodynamic parameters, physician-level practice variations). Future multi-center studies incorporating more detailed clinical data on resuscitation protocols and physician decision-making are needed to validate our findings and address these limitations.

## 6 Conclusion

Albumin infusion demonstrates no survival advantage in patients with SIMI. These findings reinforce crystalloids as the evidence-based fluid of first choice in initial resuscitation strategies within contemporary management protocols.

## Data Availability

Publicly available datasets were analyzed in this study. This data can be found here: https://physionet.org/content/mimiciv/ (accession number: MIMIC-IV, version 2.2).

## Glossary

SIMI: sepsis-induced myocardial injury
ICU: intensive care unit
KM: Kaplan-Meier
PSM: propensity score matching
MIMIC-IV: Medical Information Mart for Intensive Care IV
SOFA: Sequential Organ Failure Assessment
T: temperature
RR: respiratory rate
SBP: systolic blood pressure
DBP: diastolic blood pressure
MAP: mean arterial pressure
HR: heart rate
SpO2: oxygen saturation
cTnT: serum cardiac troponin T
WBC: white blood count
PLT: platelet count
BUN: blood urea nitrogen
PT: prothrombin time
COPD: chronic obstructive pulmonary disease
CKD: chronic kidney disease
APSIII: Acute Physiology Score III
SOFA: Sequential Organ Failure Assessment
e-GFR: estimated Glomerular Filtration Rate
CRRT: continuous renal replacement therapy
IMV: invasive mechanical ventilation
LOS: length of stay
IQR: interquartile range
SMD: standardized mean differences
eGC: endothelial glycocalyx
HR: hazard ratio
CI: confidence interval
OR: odds ratio
SD: standard deviation
IQR: interquartile range
