# Supplementary material for: Albumin administration and 28-day mortality in sepsis-induced myocardial injury: a propensity score-matched analysis
Source: Front Pharmacol. 2026 Jun 30;17:1844892. doi: 10.3389/fphar.2026.1844892 (PMC13365149; doi:10.3389/fphar.2026.1844892)
Supplement: Supplementary file 1 [file DataSheet1.PDF]

Supplementary Material

1. Supplementary Tables

Table A1: Handling of missing/outliers

Variable	Missing number	Percent (%)	Handling method	Outlier number	Percent(%)	Handling method
Albumin	-	-	-	1	0.06	Replace with the median
Hemoglobin	1	0.06	Replace with the mean	-	-	-
Cr	7	0.4	-	-	-	-
T	1	0.06	Replace with the median	13	0.8	winsor2 (0.5 99.5)
WBC	4	0.2	Replace with the median	13	0.8	winsor2 (0.5 99.5)
PLT	7	0.4	Replace with the median	-	-	-
PT	99	6.3	Regression imputation	-	-	-
Glucose	-	-	-	13	0.8	winsor2 (0.5 99.5)
BUN	4	0.2	Replace with the median	-	-	-
Lactate	207	13.3	Regression imputation	-	-	-
RR	-	-	-	19	1.2	winsor2 (1 99.5)

SBP	-	-	-	11	0.7	winsor2 (0.5 99.5)
DBP	-	-	-	20	1.3	winsor2 (1 99.5)
MAP	-	-	-	21	1.3	winsor2 (0.5 99)

Table A2: Cox regression analysis after PSM

Variable	Hazard Ratio	Lower CI	Upper CI	P value
Age	1.019925524	1.004095656	1.036004954	0.013432311
Lactate	1.067640295	0.985960732	1.156086406	0.107008748
WBC	1.002229056	0.975917586	1.029249903	0.869701605
Albumin infusion	0.85348075	0.546287661	1.333417251	0.486453837
Hemoglobin	0.898895345	0.810362923	0.997099963	0.043918952
PLT	0.996777235	0.994581729	0.998977587	0.004114991
PT	1.022655436	1.001184491	1.044586838	0.038517037
Glucose	0.99906495	0.99571742	1.002423734	0.584863508
Cirrhosis	1.821128099	1.162183154	2.85368751	0.008901258
CKD	0.719729769	0.425921651	1.216211807	0.219190887
COPD	0.928255777	0.40345799	2.135684034	0.860986078
DM	0.571267515	0.341072668	0.956824174	0.033363191
Temperature	0.81601281	0.663570989	1.003475011	0.053968981

Table A3: Albumin mortality impact via PSM-adjusted survival analysis post lactate/PT exclusion

	Non albumin group	Albumin group	P value
28-day mortality			
Model1	26/308	26/308	0.26
Model2	26/298	28/298	0.36
60-day mortality			
Model1	32/308	36/308	0.21
Model2	33/298	38/298	0.22
90-day mortality			
Model1	34/308	38/308	0.17
Model2	35/298	40/298	0.16
In-hospital mortality			
Model1	36/308	39/308	0.09
Model2	36/298	40/298	0.12

Model1: Effects of albumin infusion and mortality after PSM without lactate values missing data; Model2: Effects of albumin infusion and mortality after PSM without lactate and PT values missing data. Mortality was expressed as deaths per total patients (deaths/total), and between-group differences were analyzed using log-rank testing (reported P-values).

Table A4: Logistic regression assessing albumin infusion's mortality effect post-lactate/PT multiple imputation

	OR	CI	P value
28-day mortality	1.07	0.66-1.72	0.791
60-day mortality	1.17	0.76-1.79	0.482
90-day mortality	1.19	0.78-1.82	0.412
In-hospital mortality	1.20	0.79-1.82	0.399

2. Supplementary Figures

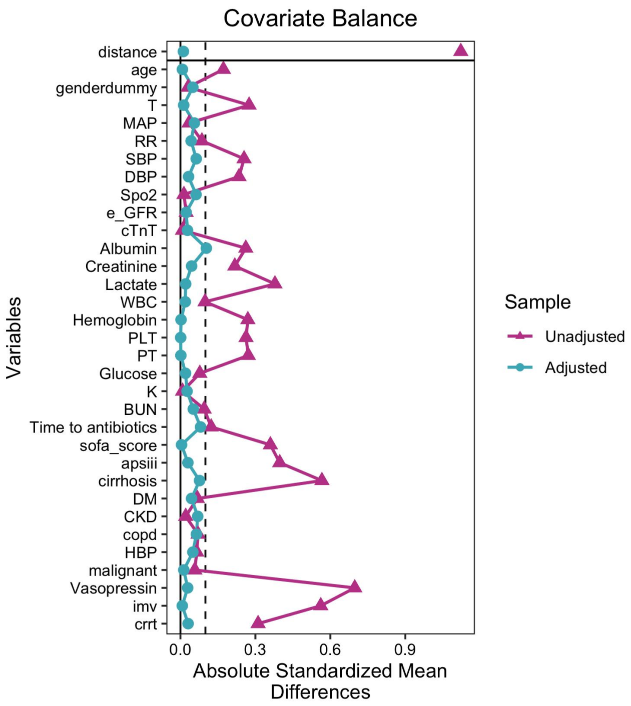

Supplementary Figure 1. Covariate Balance Before and After Propensity Score Matching: Non-Albumin vs. Albumin

Note: We performed 1:1 propensity score matching with a caliper width of 0.05 standard deviations to compare non-albumin vs. albumin administration. After matching, standardized mean differences (SMD) for all covariates were most below 0.1.

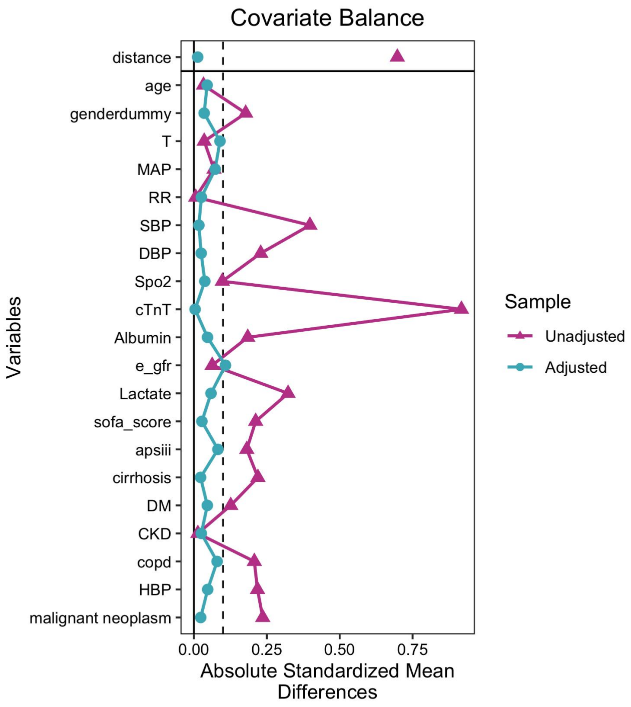

Supplementary Figure 2. Covariate Balance Before and After Propensity Score Matching: Early Albumin vs. Late Albumin

Note: We performed 1:2 propensity score matching with a caliper width of 0.1 standard deviations to compare early vs. late concentration albumin administration. After matching, standardized mean differences (SMD) for all covariates were most below 0.1.

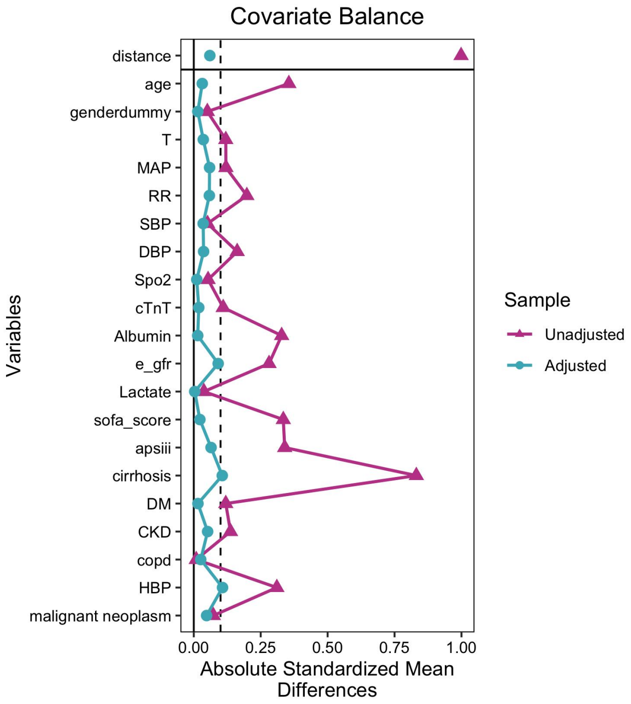

Supplementary Figure 3. Covariate Balance Before and After Propensity Score Matching: Low vs. High Concentration Albumin

Note: We performed 1:1 propensity score matching with a caliper width of 0.1 standard deviations to compare low vs. high concentration albumin administration. After matching, standardized mean differences (SMD) for all covariates were most below 0.1.

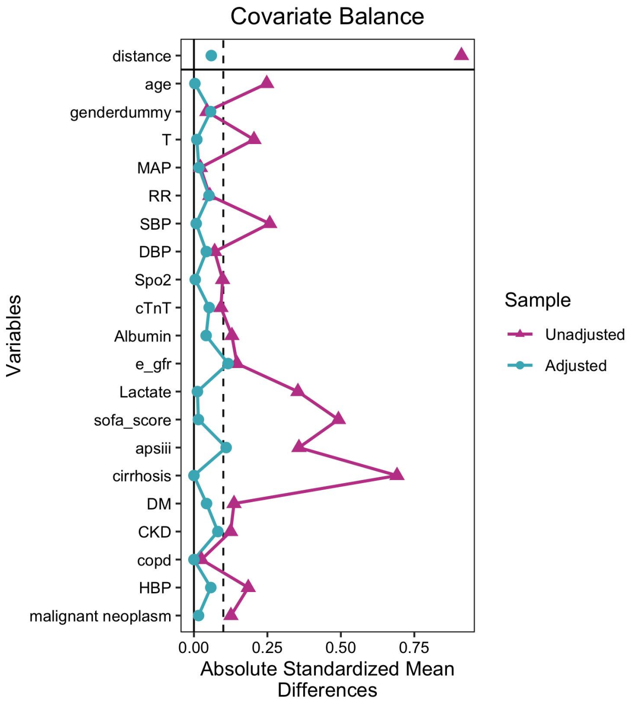

Supplementary Figure 4. Covariate Balance Before and After Propensity Score Matching: Low vs. High Dose Albumin

Note: We performed 1:1 propensity score matching with a caliper width of 0.1 standard deviations to compare low vs. high dose albumin administration. After matching, standardized mean differences (SMD) for all covariates were most below 0.1.
